# Dissecting Psychiatric Heterogeneity and Comorbidity with Core Region-Based Machine Learning

**DOI:** 10.1007/s12264-023-01057-2

**Published:** 2023-04-24

**Authors:** Qian Lv, Kristina Zeljic, Shaoling Zhao, Jiangtao Zhang, Jianmin Zhang, Zheng Wang

**Affiliations:** 1 School of Psychological and Cognitive Sciences, Beijing Key Laboratory of Behavior and Mental Health, IDG/McGovern Institute for Brain Research, Peking-Tsinghua Center for Life Sciences, Peking University, Beijing, 100871 China; 2 School of Health and Psychological Sciences, City, University of London, London, EC1V 0HB UK; 3 Institute of Neuroscience, State Key Laboratory of Neuroscience, CAS Center for Excellence in Brain Science and Intelligence Technology, Chinese Academy of Sciences, Shanghai, 200031 China; 4 University of Chinese Academy of Sciences, Beijing, 101408 China; 5 Tongde Hospital of Zhejiang Province (Zhejiang Mental Health Center), Zhejiang Office of Mental Health, Hangzhou, 310012 China; 6 School of Biomedical Engineering, Hainan University, Haikou, 570228 China

**Keywords:** Psychiatric disorders, Obsessive-compulsive disorder, Core region, Magnetic resonance imaging, Machine learning, Neuroimaging-based diagnosis

## Abstract

Machine learning approaches are increasingly being applied to neuroimaging data from patients with psychiatric disorders to extract brain-based features for diagnosis and prognosis. The goal of this review is to discuss recent practices for evaluating machine learning applications to obsessive-compulsive and related disorders and to advance a novel strategy of building machine learning models based on a set of core brain regions for better performance, interpretability, and generalizability. Specifically, we argue that a core set of co-altered brain regions (namely ‘core regions’) comprising areas central to the underlying psychopathology enables the efficient construction of a predictive model to identify distinct symptom dimensions/clusters in individual patients. Hypothesis-driven and data-driven approaches are further introduced showing how core regions are identified from the entire brain. We demonstrate a broadly applicable roadmap for leveraging this core set-based strategy to accelerate the pursuit of neuroimaging-based markers for diagnosis and prognosis in a variety of psychiatric disorders.

## Introduction

Psychiatric heterogeneity and comorbidity are ubiquitous in the categorical diagnosis of mental disorders, and this remains a bottleneck for precision diagnosis and personalized therapy [1–4]. Obsessive-compulsive disorder (OCD) is a highly complex and disabling psychiatric disorder characterized by obsessions and/or compulsions [5, 6], with a lifetime prevalence of ~ 1% to 2% worldwide [7]. Obsessions are repetitive thoughts, images, or urges that are intrusive and cause distress or anxiety, such as fear of contamination. Compulsions are repetitive and stereotyped behaviors that patients feel driven to perform to reduce the anxiety associated with obsessions, such as washing and cleaning. The current gold standard for a clinical diagnosis of OCD relies on the symptom-based criteria of the Diagnostic and Statistical Manual of Mental Disorders [8], and the symptom severity of patients with OCD is typically quantified by behavioral assessment instruments such as the Yale-Brown Obsessive-Compulsive Scale (Y-BOCS) [9]. The need to unbolt the shackles of the multifaceted symptom dimensions that characterize OCD, as well as phenotypes that co-occur with other disorders, has led researchers to search for objective and tangible biomarkers for diagnosis and treatment prognosis in the individual [10].

Brain imaging techniques such as magnetic resonance imaging (MRI) and positron emission tomography (PET) have demonstrated their capacity for non-invasive investigation of brain structure and function in patients with OCD, leading to unprecedented advancement in the characterization of its etiology [11]. Specifically, structural and functional alterations in patients with OCD have been found in the orbitofrontal cortex (OFC), anterior cingulate cortex (ACC), dorsolateral prefrontal cortex (PFC), inferior frontal gyrus (IFG), insula, amygdala, striatum, and thalamus; these were central to the development of the traditional cortico-striato-thalamo-cortical hypothesis [5]. Different degrees and patterns of alterations in these neurocircuits in individuals with OCD are thought to lead to phenotypic heterogeneity and variable responses to pharmacotherapy and neurosurgical interventions [12–16]. To resolve the heterogeneity in OCD, psychiatrists have attempted to group patients with OCD based on clinical manifestations, and four subtypes have been consistently identified: contamination/washing, aggression/checking, symmetry/ordering, and hoarding [17]. Subsequent neuroimaging studies have confirmed the distinct neural mechanisms underlying these subtypes [18, 19]. In subjects with obsessive-compulsive traits, data-driven clustering of symptoms has revealed two subgroups with distinct symptom patterns and structural abnormalities [20]. Focused on the heterogeneity in brain imaging, recent studies have also identified subtypes of OCD with distinct neuroimaging abnormalities [21]. However, our understanding of OCD psychopathology stems predominantly from studies using case-control designs to focus on group differences between patients and healthy controls, which have proven challenging to translate into clinical utility. As such, characterizing individual structural and functional variations in the psychiatric brain has become an important prerequisite to the pursuit of translationally valuable imaging biomarkers.

**Fig. 1:**
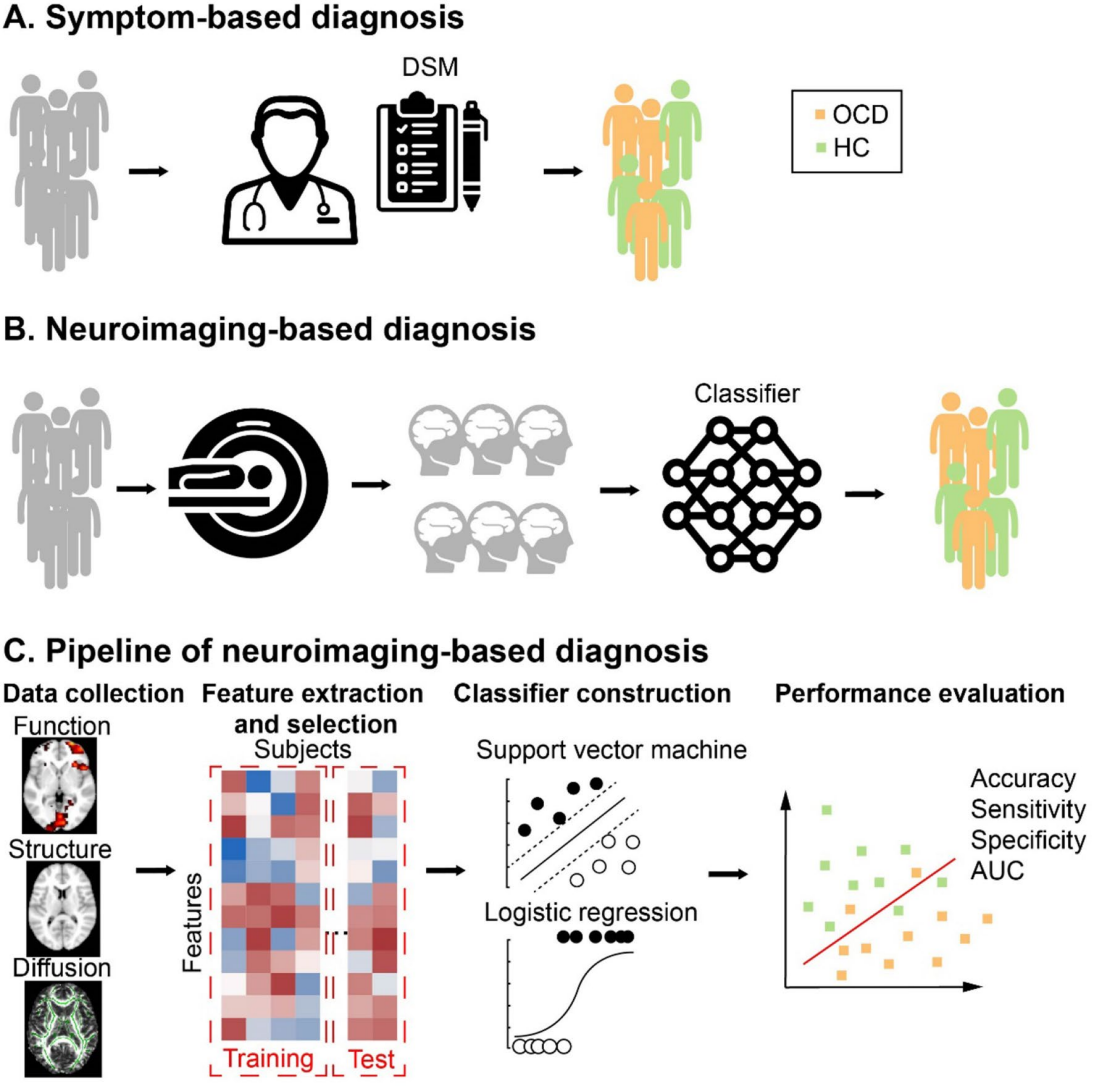
The application of neuroimaging-based diagnosis in OCD. **A**, **B** Comparison of traditional symptom-based diagnosis and neuroimaging-based diagnosis in OCD. Traditionally, patients with OCD are diagnosed by psychiatrists according to the DSM. In neuroimaging-based diagnosis, imaging datasets from subjects are acquired, and a classifier automatically distinguishes patients with OCD from HCs. **C** The pipeline of diagnostic classification based on neuroimaging data. DSM, the Diagnostic and Statistical Manual of Mental Disorders; OCD, obsessive-compulsive disorder; HC, healthy control; AUC, the area under the curve.

Machine learning algorithms are increasingly being combined with neuroimaging techniques to make inferences about the health status of subjects at the individual level, thereby enabling the automatic and objective diagnosis of psychiatric disorders [22] (Fig. 1). This technique has been used in numerous recent investigations to distinguish patients with OCD from healthy controls (HCs) [23]. Here, we will briefly introduce the pipeline for these machine-learning techniques; further technical details can be found in several excellent recent reviews [22].

Although specific procedures might vary at different stages across studies, the typical analysis pipeline includes the following steps. The first step is to transform neuroimaging data into features. This involves deciding which features to use and extracting feature values from the data. The term ‘feature’ refers to any derived variable containing valuable information about the class labels that can be extracted from the data. Different types of features in neuroimaging data can be used for classification purposes. For example, features can be extracted at the voxel level, or based on pre-defined areas (regions of interest) from a structural or functional brain atlas. Alternatively, multiple voxels across a brain network can be combined using dimension reduction techniques or joint estimation of multimodal features. Once features have been extracted from a training dataset, the number of features is most often reduced either using a data-driven method or based on prior knowledge [24]. Next, the selected features are fed into the machine learning algorithms to learn from the labeled data. A classifier is a function that takes features as input and generates a class label prediction. Popular machine learning classifiers for mental disorders include the support vector machine (SVM), logistic regression (LR), and random forest approaches [25]. Constructed classifiers require validation to determine model performance and generalizability, for which k-fold cross-validation (CV) is widely-used. To mitigate overfitting and performance bias, it is critical to analyze training and testing datasets independently. In k-fold CV, the most parsimonious way is to evenly divide the complete dataset into *k* test sets: k − 1 sets combined for model training, and one left-out set for testing. The average performance gives an approximation of the quality of models generated with this training algorithm. When the sample size is small, a special form of k-fold CV known as leave-one-out cross-validation (LOOCV) is typically adopted, where k is the total number of subjects. Finally, classification performance can be evaluated with metrics such as accuracy, specificity, sensitivity, and the area under the curve (AUC) of the receiver operating characteristic (ROC) curve. These metrics evaluate different aspects of classifier performance. Accuracy reflects how many cases were correctly classified out of the total (patients and HCs). Meanwhile, sensitivity and specificity refer to the proportion of correctly identified patients and HCs, respectively. The ROC curve is commonly used to demonstrate the trade-off between sensitivity and specificity, with a larger AUC of the ROC corresponding to the better overall performance of the classifier.

Despite their widespread use, current machine-learning models face a number of notorious challenges that require further consideration. One such challenge is that the number of features in a neuroimaging dataset is typically several orders of magnitude larger than the number of subjects, namely, the curse of dimensionality. Effective feature engineering can therefore significantly reduce the computational load and improve efficiency and performance [26, 27], particularly by taking advantage of the neurocircuitry characteristics of brain diseases [28]. The consensus on what constitutes pathological circuits underlying psychiatric disorders like OCD is that widespread structural and functional alterations over the whole brain are implicated whereas not all brain regions are uniformly involved in the etiology of OCD [6, 29]. Screening key brain-based features to separate them from less relevant features is both crucial and beneficial. Here, we review studies relevant to the diagnostic classification of OCD to underscore the contribution of core pathological regions. We then propose a novel classification strategy based on features from a set of core brain regions, and evidence endorsing this concept is discussed. A wide range of methods that aid in screening these core regions out of the whole brain is discussed. Finally, we outline future directions to accelerate the pursuit of imaging biomarkers in the diagnosis and prognosis of OCD.

## Diagnostic Biomarkers for OCD

In this review, we concentrate on MRI modalities as they have been most widely applied in the machine-learning literature related to OCD [30]. In July 2022, a systematic literature search was done using PubMed with the following inclusion criteria: (1) MRI data were acquired; (2) a machine learning algorithm was used to classify patients with OCD and HCs; (3) the article was published in a peer-reviewed journal; (4) the article was in English. This search resulted in 24 relevant studies which are reviewed here [31–54]. Data from three MRI modalities were analyzed in these studies: structural (6 articles), functional (16 articles), and diffusion (1 article) MRI. Only one study used both structural and diffusion imaging data [40], but the combination of any two modalities in one classifier was not explored. See Table 1 for a summary of these studies and the methods used.

**Table 1:** Diagnostic classification of OCD.

References	Modality	Sample size	Classifier	Validation methods	Key features	Overall Performance
Soriano-Mars *et al.* [47]	Structural MRI	OCD = 72/30, HC = 72/30	Not specified	External validation	Gray matter volume in OFC/medial PFC, posterior cingulate cortex/precuneus, cerebellum, posterior insula, ventral striatum, and thalamus	Accuracy 76.6%, sensitivity 70.0%, specificity 83.3%
Weygandt *et al.* [48]	Task fMRI	OCD = 10, HC = 10	SVM	LOOCV	Discriminative volume decoding affective pictures in OFC and caudate	Accuracy 100.0%
Gruner *et al.* [41]	Resting-state fMRI	OCD = 23, HC = 23	LR	LOOCV	ICA components of the middle frontal/dorsal anterior cingulate network, the anterior/posterior cingulate network, and the visual network	Accuracy 80.1%
Li *et al.* [42]	Diffusion MRI	OCD = 28, HC = 28	SVM	LOOCV	FA in the prefrontal and temporal white matter, inferior fronto-occipital fasciculus, superior fronto-parietal fasciculus, splenium of the corpus callosum, and middle cingulum bundle	Accuracy 84.0%, sensitivity 86.0%, specificity 82.0%
Parrado-Hernandez *et al.* [45]	Structural MRI	OCD = 86, HC = 86	SVM	LOOCV	GMV in the OFC, PFC, ACC, insula, temporal and parietal cortices, and subcortical structures like the caudate, putamen, and thalamus	Accuracy 73.8%, sensitivity 74.1%, specificity 73.6%
Yun *et al.* [50]	Structural MRI	OCD = 56, HC = 75	SVM	LOOCV	Structural covariance from cortical surface area and cortical thickness	Accuracy 90.7–95.6%, sensitivity 90.8–96.2%, specificity 91.1–95.0%
Hu *et al.* [36]	Structural MRI	OCD = 33, HC = 33	SVM, Gaussian processes classifier	LOOCV	White matter volume in the occipital, inferior parietal, precentral, postcentral, paracentral, inferior temporal, and middle frontal cortices	Accuracy 81.8%, sensitivity 87.9%, specificity 75.8%
Takagi *et al.* [52]	Functional MRI	OCD = 56/10, HC = 52/18	Sparse LR	External validation	Functional connectivity in the frontoparietal and default mode networks	AUC 0.70
Trambaiolli *et al.* [44]	Structural MRI	OCD = 38, HC = 36	SVM	LOOCV	Volume of the caudate, putamen, IFG, precentral gyrus, fusiform gyrus, and lateral OFC	Accuracy 71.6%
Zhou *et al.* [40]	Structural and diffusion MRI	OCD = 48, HC = 45	SVM	LOOCV	FA in the uncinate fasciculus, the cingulum in the hippocampus, corticospinal tract, and cerebral peduncle	Accuracy 80.7%, sensitivity 81.2%, specificity 80.0%, AUC 0.83
Bu *et al.* [53]	Resting-state fMRI	OCD = 54, HC = 54	SVM	LOOCV	ALFF in ventromedial PFC, dorsolateral PFC, insula, inferior parietal lobule, and occipital lobe	Accuracy 95.4%, sensitivity 96.3%, specificity 94.4%, AUC 0.99
Hu *et al.* [37]	Resting-state fMRI	OCD = 88, HC = 88	SVM	LOOCV	ReHo in the OFC, dorsal ACC, inferior parietal cortex, temporal regions, and cerebellum	Accuracy 79.0%, sensitivity 78.4%, specificity 79.6%
Yang *et al.* [33]	Resting-state fMRI	OCD = 68, HC = 68	SVM	LOOCV	fALFF in the superior temporal gyrus, middle temporal gyrus, supramarginal gyrus, and superior parietal lobule	Accuracy 72.0%, sensitivity 68.0%, specificity 76.0%
Bruin *et al.* [54]	Structural MRI	OCD = 2304, HC = 2068	Multiple algorithms	Inter-site CV	No consistent features	Accuracy ~50.0%, sensitivity ~50.0%, specificity ~50.0%, AUC ~0.50
Jia *et al.* [39]	Resting-state fMRI	OCD = 40, HC = 38	SVM	LOOCV	voxel-mirrored homotopic connectivity in the thalamus and postcentral gyrus	accuracy 94.9%, sensitivity 95.0%, specificity 94.7%
Liu *et al.* [35, 51]	Resting-state fMRI	OCD = 50, HC = 50	SVM	LOOCV	Effective connectivity and functional connectivity associated with the frontal-parietal cortex, basal ganglia, and cerebellum	Accuracy 80.5%
Sen *et al.* [49]	Resting-state fMRI	OCD = 15, HC = 13	SVM	LOOCV	Edge entropy connected with the frontal lobe, parietal lobe, ACC, posterior cingulate gyrus, thalamus, default mode network, NAc, and amygdala	Accuracy 89.1%, sensitivity 80.0%, specificity 100.0%
Xing *et al.* [34]	Resting-state fMRI	OCD = 61, HC = 67	Random forest	LOOCV	Intra-cerebellar connectivity, connectivity between the cerebellum and basal ganglia, connectivity between the rectus and parahippocampal gyrus, and corticothalamic connectivity	Accuracy 91.8%, sensitivity 92.6%, specificity 90.7%
Zhan *et al.* [31]	Resting-state fMRI	OCD = 92, HC = 79	Sparse LR	Cross-species LOOCV	Functional connectivity with the central temporal cortex, superior temporal cortex, dorsolateral PFC, primary somatosensory cortex, primary motor cortex, ACC, centrolateral PFC, superior parietal cortex, and ventrolateral PFC	78.4%, sensitivity 73.9%, specificity 83.5%, AUC 0.85
Luo *et al.* [38]	Resting-state fMRI	OCD = 61, HC = 67	SVM	10-fold CV	Distance correlations distributed in the dorsolateral PFC, orbital part of superior frontal gyrus, middle frontal gyrus, ACC, paracingulate gyri, supplementary motor area, and precuneus	Accuracy 93.0%, sensitivity 95.1%, specificity 89.7%
Liu *et al.* [43]	Resting-state fMRI	OCD = 73, HC = 73	SVM	10-fold CV	Dynamic ALFF in the inferior parietal lobule, dorsolateral PFC, middle occipital gyrus, and cuneus	Accuracy 83.6%, sensitivity 80.8%, specificity 86.3%
Kalmady *et al.* [32]	Resting-state fMRI	OCD = 175, HC = 175	LR	5-fold CV	Six fMRI features in the default mode, language, attention, visual, auditory, motor, and salience networks	Accuracy 80.3%, sensitivity 82.7%, specificity 77.8%, precision 79.2%
Yang *et al.* [46]	Resting-state fMRI	OCD = 62, HC = 65	SVM	10-fold CV	Functional connectivity with the OFC, IFG, insula, middle cingulate cortex, putamen, thalamus, temporal cortices, and parahippocampal cortex	Accuracy 87.6%, sensitivity 95.2%, specificity 83.6%, AUC 0.89

MRI, magnetic resonance imaging; fMRI, functional magnetic resonance imaging; OCD, obsessive-compulsive disorder; HC, healthy control; SVM, support vector machine; LOOCV, leave-one-out cross-validation; LR, logistic regression; CV, cross validation, AUC, area under the curve; OFC, orbitofrontal cortex; PFC, prefrontal cortex; ICA, independent component analysis; FA, fractional anisotropy; GMV, gray matter volume; ACC, anterior cingulate cortex; NAc, nucleus accumbens; IFG, inferior frontal gyrus; ALFF, amplitude of low-frequency fluctuations; fALFF, fractional amplitude of low-frequency fluctuations; ReHo, regional homogeneity.

### Functional MRI

To date, only one study [48] has used a task-based functional MRI dataset to distinguish patients with OCD from HCs (OCD = 10, HC = 10). Affective pictures from three categories were presented during functional MRI scanning. A two-step pattern recognition analysis was conducted to classify subjects into two groups. They trained a linear support vector classifier to distinguish the picture categories based on the subject’s brain response, and a searchlight method was then applied to predict the diagnostic label of the subjects with information from the first step. The predictive power of features extracted from the OFC and the caudate nucleus even reached close to 100.0% accuracy, a finding that certainly requires future validation with multi-site and large-scale external data.

Since the pilot work of Biswal *et al.* [55], resting-state functional MRI has become a popular technique to investigate functional dysfunction in psychiatric disorders [56, 57]. Multiple measures derived from resting-state fMRI data have been used as candidate features trained for the diagnosis of OCD, such as the functional connectivity, the amplitude of low-frequency fluctuations (ALFF), the fractional amplitude of low-frequency fluctuations (fALFF), and regional homogeneity (ReHo). Gruner *et al.* [41] were the first to perform diagnostic classification for OCD based on 36 functional networks decomposed from resting-state functional data. The assembly of three networks combined with the LR algorithm achieved the highest performance at an accuracy of 80.1%, including the middle frontal/dorsal anterior cingulate network, the anterior/posterior cingulate network, and the visual network. Hu *et al.* [37] focused on ReHo maps to train SVM classifiers in tandem with LOOCV achieving 79.0% accuracy for patients with OCD (sensitivity 78.4%, specificity 79.6%). The key regions contributing to the SVM classifier were the right OFC, dorsal ACC, inferior parietal cortex, temporal regions, and cerebellum, among which the right OFC exhibited the highest discriminative power. Using features derived from the fALFF map, Yang *et al.* [33] applied SVM to discriminate 68 drug-naïve patients with OCD from 68 HCs. The overall accuracy was 72.0% with 68.0% sensitivity and 76.0% specificity. Brain regions contributing to the discrimination consisted of the left superior temporal gyrus, the right middle temporal gyrus, the left supramarginal gyrus, and the superior parietal lobule. Jia *et al.* [39] adopted the voxel-mirrored homotopic connectivity method to investigate interhemispheric coordination in OCD. Patients with OCD showed significantly decreased homotopic connectivity in the OFC, thalamus, middle occipital gyrus, precentral gyrus, and postcentral gyrus compared with HCs. Exploratory SVM was performed with homotopic connectivity indices based on these five brain regions and all possible pairwise combinations. And they found that a combination of the thalamus and postcentral gyrus achieved the highest accuracy of 94.9%. In drug-naïve patients with OCD, Bu *et al.* [53] tested a wide range of imaging features extracted from ALFF, fALFF, ReHo, and functional connectivity strength, and found that the SVM classifier yielded the highest accuracy of 95.4% with ALFF extracted from the PFC, ACC, precentral gyrus, and occipital lobes. Liu *et al.* [35, 51] conducted a series of studies to distinguish medication-free patients from HCs with effective connectivity and functional connectivity. The combined classifier outperformed the classifiers with any type of connectivity (80.5% accuracy). To reduce the number of features extracted from whole-brain functional connectivity, Takagi *et al.* [52] applied principal component analysis to reduce the number of features from nearly 10,000 down to the number of participants. An additional feature selection procedure, canonical correlation analysis, was applied to extract smaller sets of features related to diagnosis. The selected features were then entered into a sparse LR model, and the LOOCV achieved an accuracy of 73.0% in the training dataset. They further tested the classifier in an external dataset (OCD = 10, HC = 18), and the AUC of the classifier was 0.7.

In addition, new functional measures were developed to expand the application of functional data in diagnostic classification. Liu *et al.* [43] applied a sliding-window approach to extract the dynamic ALFF as features in 73 OCD and 73 HCs. The SVM classifier with 10-fold CV successfully distinguished patients (83.6% accuracy, 80.8% sensitivity, and 86.3% specificity) from HCs with the most discriminative regions located in the inferior parietal lobule, dorsolateral PFC, middle occipital gyrus, and cuneus. Luo *et al.* [38] used distance correlation to construct the functional connectivity matrices (OCD = 61, HC = 67), and the best discriminative features were selected by SVM recursive feature elimination with a 10-fold CV strategy. The features from distance correlation achieved an accuracy of 93.0% (89.7% specificity and 95.1% sensitivity), superior to features from either Pearson correlation or partial correlation. The most discriminative features from distance correlation were distributed in the right dorsolateral PFC, orbital part of left superior frontal gyrus and right middle frontal gyrus, right ACC, paracingulate gyri, left supplementary motor area, and right precuneus.

Recent developments along this line of research focus on new machine-learning models to boost classification performance [31, 34, 49]. In a small dataset (OCD = 15, HC = 13), Sen *et al.* [49] introduced an information-theoretic approach to extract features from resting-state data and found that the features derived from the differential sub-graph (edge) entropy achieved better performance (89.1% accuracy, 100.0% specificity, and 80.0% sensitivity) than other feature engineering methods. The most discriminative features were located in the frontal lobe, parietal lobe, ACC, posterior cingulate gyrus, thalamus, default mode network, nucleus accumbens (NAc), and amygdala. Using whole-brain functional connectivity matrices, Xing *et al.* [34] put forward a new Riemann Kernel principal component analysis for feature extraction. The proposed feature selection algorithm combined with an XGBoost classifier yielded the highest accuracy (91.8%). The decisive features were found in intra-cerebellar connectivity, connectivity between the cerebellum and basal ganglia, connectivity between the rectus and parahippocampal gyrus, as well as corticothalamic connectivity. Yang *et al.* [46] integrated deep learning with traditional machine learning in one framework to distinguish 62 OCD from 65 HCs. A novel spatial similarity-aware learning model was proposed to construct the functional connectivity matrix, and a fused deep polynomial network model was subsequently applied to learn informative features. The SVM classifier with these informative features yielded the best performance (87.6% accuracy, 95.2% sensitivity, and 83.6% specificity) among features extracted from other types of functional connectivity and other feature learning methods. The most effective features were connected with OFC, IFG, insula, middle cingulate cortex, putamen, thalamus, temporal cortices, and parahippocampal cortex. Kalmady *et al.* [32] predicted the diagnosis of OCD (OCD = 175, HC = 175) with an established framework called EMPaSchiz, which was proposed to extract both regional and connectivity-based features onto 14 different brain parcellation schemes in schizophrenia [58]. The EMPaSchiz algorithm with an LR classifier was able to predict OCD with 80.3% accuracy using the 5 times 5-fold CV, outperforming the neural network model. The most important features were distributed in several functional brain networks, such as the default mode, language, attention, visual, auditory, motor, and salience networks. Using a cross-species strategy, a recent study transferred the features extracted from transgenic monkeys to discriminate HCs from patients in several psychiatric disease cohorts, including autism spectrum disorder (ASD), OCD, and attention deficit hyperactivity disorder [31]. A set of core brain regions distributed in the frontal and temporal cortices was extracted from the monkey dataset and then used to construct a new classifier to classify human patients in multiple independent clinical cohorts. In the OCD cohort, the monkey-derived classifier achieved an average accuracy of 78.4%.

### Structural MRI

As a clinically valuable tool, MRI measurement has been applied to non-invasively quantify the morphological structure of the gray and white matter in psychiatric patients. In 2007, Soriano-Mas *et al.* [47] applied voxel-wise *t*-tests to compare the regional gray matter volume of the whole brain in a cohort (OCD = 72, HC = 72) and defined the individual expression value as the scalar product of an individual volumetric map of gray matter by the *t* map of group differences between OCD and HC. Next, the Euclidean distances between individual expression values and each group’s mean expression value were used as candidate features to train a classifier for the diagnostic classification of OCD. The classifier yielded an accuracy of 93.1% in the training cohort with LOOCV, while the overall accuracy was only 76.6% in an independent cohort (OCD = 30, HC = 30). The most relevant features were found in the OFC/medial PFC, the posterior cingulate cortex/precuneus, cerebellum, posterior insula, ventral striatum, and thalamus. Trambaiolli *et al.* [44] aimed to distinguish 38 patients with OCD from 36 HCs using the volumetric data of 117 cortical and subcortical regions. All 117 features were submitted to 7 kinds of feature selection algorithms and trained with SVM classifiers in a LOOCV fashion. Interestingly, the *t*-test feature selection method yielded the best performance, with an average accuracy of 71.6%. The most discriminative and consistent features were found in the left caudate, bilateral putamen, bilateral IFG, left precentral gyrus, right fusiform gyrus, and right lateral OFC. Parrado-Hernández *et al.* [45] proposed a new feature selection method for classification with structural MRI data in OCD. To identify stable features, voxel-wise features were selected by training an ensemble of linear classifiers based on randomly selected GMV data. In their cohort (OCD = 86, HC = 86), these features achieved an accuracy of 73.8% with 74.1% sensitivity and 73.6% specificity. The stable features contributing to the classification were widely distributed in the brain, including the OFC, PFC, ACC, insula, temporal, and parietal cortices, as well as subcortical structures like the caudate, putamen, and thalamus.

In some studies, many types of structural measures have been used to gather more information. Based on the treatment response to pharmacotherapy, Yun *et al.* [50] divided the patients with OCD into responders (*n* = 25) and non-responders (*n* = 31). Cortical thickness and cortical surface area before treatment were estimated from structural MRI data and used to construct pair-wise structural covariance networks in individuals. A univariate *t*-test was applied in a leave-one-out manner to identify the significant differences in the structural covariance network as features. The SVM classifiers were trained to successfully differentiate HCs from responders or non-responders among patients with OCD (OCD responders *versus* HC: 95.6% accuracy, OCD non-responders *versus* HC: 90.7% accuracy). Hu *et al.* [36] used both gray matter and white matter volume data for OCD classification. Both SVM and Gaussian process classifiers were trained to examine the classification performance of different tissue types. The best classification performance (81.8% accuracy) was achieved by the SVM classifier with white matter volumetric data, and white matter regions with superior discriminative power were located in the middle frontal gyrus, inferior parietal gyrus, inferior temporal gyrus, precentral and postcentral gyri, and occipital cortices. With both structural and diffusion MRI datasets obtained from a cohort of 48 patients with OCD from 45 HCs, Zhou *et al.* [40] fed four types of features (gray matter volume, white matter volume, fractional anisotropy, and mean diffusivity) into the SVM classifiers. The classification performance of features extracted from diffusion MRI was better than that from structural MRI. The most discriminative regions for classification were located in the angular gyrus, ACC, paracentral lobule, inferior parietal gyrus, IFG, and cerebellum.

Notably, an international OCD working group within the Enhancing Neuro-Imaging Genetics Through Meta-Analysis (ENIGMA) was initiated to aggregate structural MRI data of OCD around the world [59, 60]. Using large-scale data from ENIGMA (OCD = 2304, HC = 2608), Bruin *et al.* [54] extracted cortical thickness, surface area, and subcortical volume as classification features and submitted them to multiple machine learning models, but achieved relatively poor performance after rigorous cross-validation procedures. When the inter-site cross-validation procedure was implemented, classification performance acutely dropped to chance level.

### Diffusion MRI

A variety of metrics derived from diffusion MRI data have been used as classification features to discriminate OCD from HCs. For instance, Li *et al.* [42] applied fractional anisotropy to train the SVM classifier and identified patients with 84.0% accuracy (86.0% sensitivity, 82.0% specificity). The white matter regions contributing to classification were mainly located in the bilateral prefrontal and temporal white matter, inferior frontal-occipital fasciculus, superior frontoparietal fasciculus, splenium of the corpus callosum, and left middle cingulum bundle. In a cohort of 93 subjects (OCD = 48 and HC = 45), Zhou *et al.* [40] found that the classification performance of SVM with fractional anisotropy was better than that with mean diffusivity (80.7% *versus* 77.4%). The most discriminative fiber tracts included the uncinate fasciculus, corticospinal tract, inferior cerebellar peduncle, superior cerebellar peduncle, cingulum, pontine crossing tract, and cerebral peduncle.

### Current Status

All reviewed papers reported successful classification of patients with OCD and HCs, with accuracies ranging from 50.0% to 100.0%. Although the performance of diagnostic classification in a small population is promising, it has been suggested that studies of this kind often suffer lower generalizability [22]. Moreover, due to the limited sample size, the majority of investigations implemented the LOOCV method to train classifiers, and very few studies validated their models with external data [47, 52, 54]. On the other hand, despite the largest sample size available in the ENIGMA-OCD consortium, the overall classification accuracy was far from the diagnostic criterion, which indicated the effects of confounding factors like personal medication status on classification performance. In addition to potential technical issues that could contribute to poor performance in cohorts with large sample sizes [22], we believe that suboptimal strategies of feature engineering in MRI-based brain connectomics remain a key obstacle to unleashing the power of machine learning models for psychiatric diagnosis.

## Screening Core Regions in OCD

### The Concept of Core Regions and Relevant Evidence in OCD

Here we propose a novel machine-learning framework based on a set of core regions for diagnostic classification. Only a subset of brain regions (not all regions) indispensable to the neuropathology of brain diseases like OCD is considered as core regions. We argue that feature engineering confined to a selective neural network consisting of a set of core regions, rather than the entire brain, would effectively improve efficacy and achieve superior performance, substantially reducing the dimensionality of candidate features by discarding regions irrelevant to the underlying psychopathology. Below we elaborate on the concept of core regions in the context of OCD etiology, which can conveniently be extended to other psychiatric disorders.

**Fig. 2:**
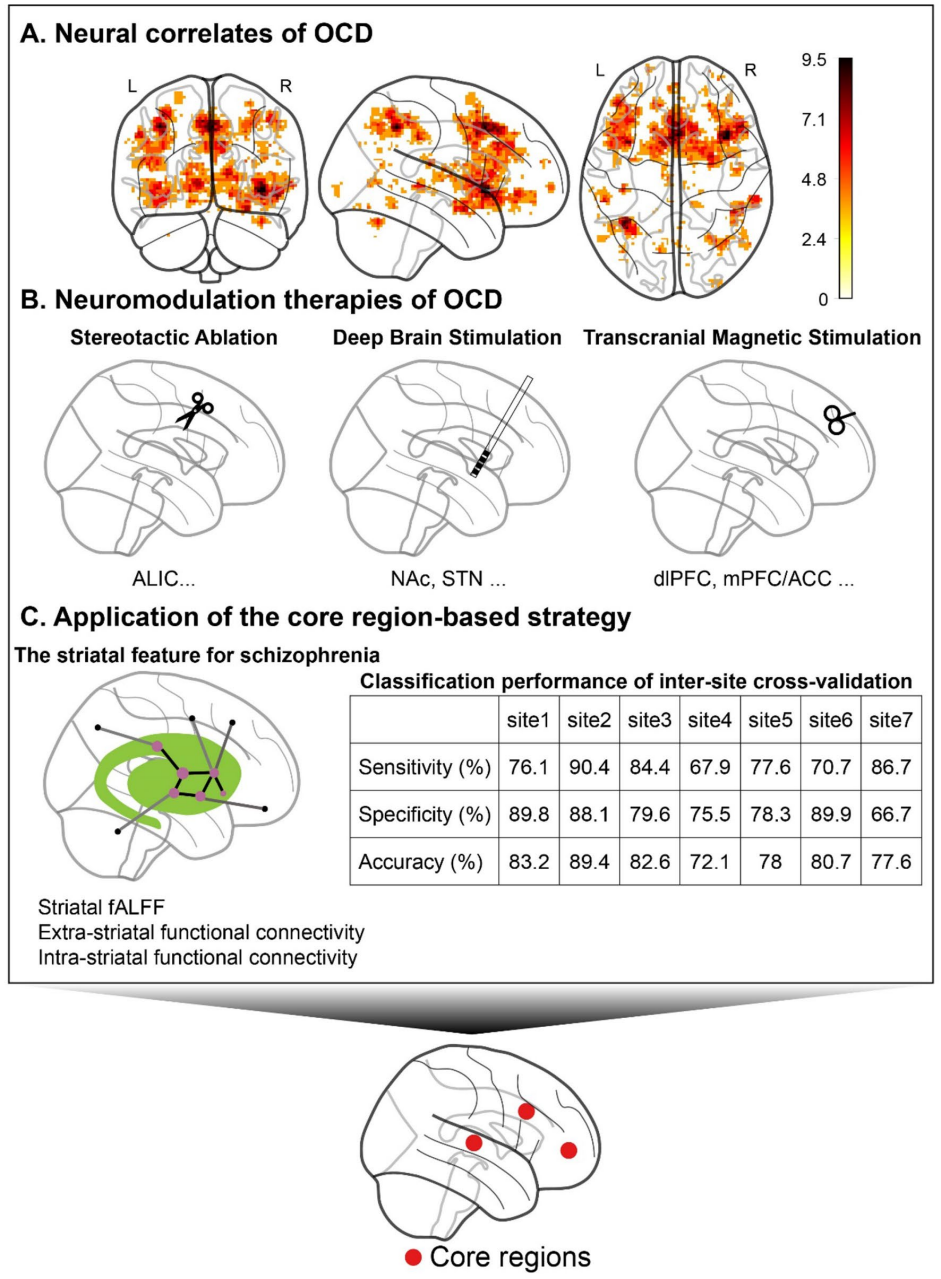
Evidence supporting the concept of core regions in OCD. **A** The Neurosynth automatically analyzed information from 81 studies investigating OCD (FDR corrected, *P <*0.01). The color bar denotes the z scores of the uniformity test. Please see the link (https://neurosynth.org/analyses/terms/ocd/) for further details. **B** Three types of neuromodulation therapies for OCD: stereotactic lesion (left), deep brain stimulation (middle), and transcranial magnetic stimulation (right). **C** Application of the core region-based strategy in schizophrenia, adapted from [101]. OCD, obsessive-compulsive disorder; ALIC, anterior limb of the internal capsule; NAc, nucleus accumbens; STN, subthalamic nucleus; dlPFC, dorsolateral prefrontal cortex; mPFC/ACC, medial prefrontal cortex/anterior cingulate cortex; FDR, false discovery rate; fALFF, fractional amplitude of low-frequency fluctuations.

Early neuroimaging studies of individuals with OCD found both structural and functional abnormalities in the OFC, ACC, caudate, and thalamus [61–67]. Dysfunction in the OFC-caudate circuit is one of the most consistently reported findings in the field [6] and appears to be clinically meaningful, with dysmetabolism in the left OFC, bilateral PFC, and ACC associated with the severity of symptoms in individual patients [64]. Recent technological developments in the fields of neuroimaging and computational psychiatry have further elucidated the roles of the IFG, ACC, insula, parietal cortex, striatum, thalamus, and cerebellum in the pathophysiology of OCD [59, 60, 68]. Moreover, different neural circuits may underlie distinct domains of neurocognitive impairment in OCD [69]. The concept of core regions relies on leveraging such findings to formulate a machine-learning model that can investigate a specific symptom dimension or behavioral domain of OCD. For instance, dysfunction in ventral cognitive circuits (composed of the IFG, ventrolateral PFC, ventral caudate, and thalamus) are likely to drive maladaptive self-regulatory behaviors in patients with OCD [70]. Put together, a core set of regions including OFC, ACC, IFG, dorsolateral PFC, insula, amygdala, striatum, and thalamus has been heavily implicated in the pathophysiology of OCD (see Fig. 2A from meta-analysis).

In treatment-resistant OCD, neurosurgical interventions such as deep brain stimulation and stereotactic ablation have been applied as a “last resort” by selectively targeting one of a few key brain regions in the frontostriatal circuits. Aside from the remarkable therapeutic effects in treated patients, these neurostimulation procedures offer valuable insights into the pathophysiological role of individual regions in the OCD circuitry.

Dorsal anterior cingulotomy and anterior capsulotomy are the most commonly used stereotactic ablative procedures in the treatment of refractory OCD [71, 72]. Cingulotomy targets the dorsal ACC and cingulum bundle, disrupting anatomical connections between the dorsal ACC, ventral striatum, and limbic structures [73]. Volumetric reductions after anterior cingulotomy have been observed in the caudate, ventral temporal-fusiform, and posterior cingulate cortex [74, 75]. Meanwhile, anterior capsulotomy targets the anterior limb of the internal capsule (ALIC), which carries bidirectional fibers connecting the frontal cortex to deep gray matter including the thalamus and basal ganglia [76–78]. Thus, a study combining structural and diffusion MRI revealed that an ALIC lesion disrupts fiber integrity in the bilateral ALIC and anterior thalamic radiation, accompanied by a decrease in gray matter volume in the PFC, ACC, striatum, thalamus, and cerebellum [13]. Furthermore, bilateral capsulotomy has been reported to alter metabolic activity in the dorsal ACC, OFC, IFG, medial dorsal thalamus, caudate, and cerebellum [79, 80]. Importantly, functional connectivity between the ventral striatum and dorsal ACC has been selectively rectified by bilateral capsulotomy in association with symptom relief in patients with OCD [16].

As a reversible alternative to surgical lesions, deep brain stimulation has demonstrated remarkable success in mitigating refractory OCD symptoms [81]. Several targets have shown safety and efficacy, including ALIC, ventral ALIC/VS (VC/VS), NAc, and subthalamic nucleus (STN) [82]. PET studies have shown that stimulation of the ALIC reduces metabolic activity in the OFC [83, 84], subgenual ACC, and right dorsolateral PFC [85]. Using diffusion imaging, studies have confirmed that the connections between the stimulation site within the ALIC and the middle frontal gyrus are associated with clinical improvement [86, 87]. Because the ALIC is also widely used in lesion procedures, it has been suggested that deep brain stimulation and lesioning exert their effects in a similar manner [88, 89]. With regard to the VC/VS target, a recent study suggested that its effective site is primarily connected to the medial OFC, dorsomedial thalamus, amygdala, and habenula [90]. Meanwhile, for the NAc-DBS, a resting-state fMRI study reported that symptom relief was correlated with the normalization of excessive connectivity between the NAc and the lateral PFC [91]. Connections with the effective site in the STN-DBS, however, include the lateral OFC, dorsal ACC, and dorsolateral PFC [90]. Furthermore, DBS in the STN has been found to reduce glucose metabolism in the OFC, medial PFC, and ACC [92].

The effectiveness of non-invasive neuromodulation techniques such as transcranial magnetic stimulation (TMS) for OCD has also been examined. However, optimal targets and stimulation frequencies for OCD treatment are still under debate in the literature. Despite some inconsistency, several recent studies have shown promising results [93]. Specifically, two studies found that active stimulation over the dorsolateral PFC resulted in significant improvement of OCD symptoms compared to sham stimulation, although the sample size was relatively small (< 20) [94, 95]. In a prospective multicenter randomized double-blind placebo-controlled trial (*n =* 99), Carmi *et al.* [96] applied high-frequency deep TMS over the medial PFC and ACC in patients with OCD who failed to respond to treatment with medications and cognitive-behavioral therapy. The reduction in the Y-BOCS was significantly greater in the active group than that in the sham group and remained significant one month after treatment. In addition, the response rate in the active group was significantly higher than that in the sham group.

Despite different targets in multiple neuromodulation therapies, all the affected brain regions converge onto the fronto-striatal-thalamic circuits (Fig. 2B) [12, 97]. Several frequently modulated key regions are also involved in the emergence of OCD symptoms, such as the OFC, dorsal ACC, dorsolateral PFC, striatum, and thalamus [63, 65, 98]. We argue on the basis of this evidence that these are core regions that are crucial in neuromodulation-mediated recovery from severe, refractory OCD symptoms.

Although the concept of core regions in diagnostic classification has not yet been formalized, several pioneering studies have already adopted classification strategies that involve selecting core regions. For example, a large body of research suggests that the striatum plays a central role in schizophrenia, with dysfunction of the striatum tightly linked to schizophrenia symptoms [99, 100]. Based on this prior knowledge, Li *et al.* [101] developed an imaging biomarker for schizophrenia, which they refer to as “functional striatal abnormalities”, that integrates information from intra- and extra-striatal functional connectivity, as well as fALFF and ReHo within the striatum. In a large multi-site cohort (*n =* 1100), the “functional striatal abnormalities” score successfully distinguished patients with schizophrenia from HCs with an overall accuracy exceeding 80.4% (79.3% sensitivity and 81.5% specificity), which is comparable to the classification performance in other large cohort studies [102–111] (Fig. 2C). In another study, Chu *et al.* [112] compared the classification performance of four feature selection methods in Alzheimer’s disease (AD), including selecting regions of interest based on prior knowledge, univariate *t*-test, recursive feature elimination, and *t*-test constrained by regions of interest. Several important regions [113] implicated in AD were selected, such as the cingulate gyrus, hippocampus, and parahippocampal gyrus. Features from these key regions (the hippocampus and parahippocampal gyrus in particular) resulted in better performance compared to features selected based on data-driven approaches such as *t*-test and recursive feature elimination. Such findings suggest that core regions can meaningfully contribute to diagnostic labels and can be used to prevent the inclusion of features that capture redundant information. Further support for this concept comes from a study done by Sheng and colleagues [114], who proposed a novel classification method to identify stages of progression toward AD, i.e., HC, mild cognitive impairment, and AD. Their algorithm achieved 89.0% accuracy in three-group classification with features from 24 regions (360 regions in total). The authors further calculated the frequency of brain regions that were selected as features, and five regions appeared more frequently than others. Features from these 5 regions successfully classified three groups (5 regions, 80.0% *versus* 24 regions, 89.0%). These findings show that the entire brain may not be necessary for effective classification, suggesting that a subset of relevant regions is sufficient for diagnostic purposes. Although no studies applied a similar strategy in patients with OCD, these examples show that this core region-based strategy can easily be applied to other mental disorders.

### Methods to Identify Core Regions in OCD

The evidence reviewed in previous sections highlights the potential of the core-region-based framework in diagnostic classification. Here we offer several methods for the selection of core regions from the whole brain to improve the diagnostic classification performance.

The roles of specific brain regions in the development and symptomology of OCD have been extensively studied and documented, resulting in rich literature. This information can be used as guidance to capture relevant features and maximize the efficiency of the classifiers. Teasing out irrelevant information is particularly important for high-dimensional neuroimaging data, which contain a large number of irrelevant and redundant features [24]. Zeng *et al.* [115] successfully applied this method by using patterns of functional connectivity to parcellate the perigenual ACC into two subregions (subgenual and pregenual) based on previous findings of dysfunction of the subgenual ACC in major depressive disorder (MDD) [116, 117] and its effectiveness as a target region for deep brain stimulation in refractory cases [118, 119]. They further used functional connectivity with these two subregions for the unsupervised classification of patients with MDD and healthy controls. Functional connectivity with the subgenual region achieved an individual‐level classification consistency of 92.5%, suggesting a core function of the subgenual ACC for the diagnostic classification of MDD. This approach has been successfully applied to other psychiatric disorders. For example, in post-traumatic stress disorder (PTSD), studies have reported abnormal resting-state functional connectivity both with the hippocampus and amygdala, and dysconnectivity with these two regions is correlated with the severity of PTSD [120]. Using functional connectivity with the amygdala as a feature, Fitzgerald *et al.* [121] demonstrated that the functional connectivity pattern with the amygdala is a reliable predictor of PTSD severity (*n =* 90, *R =* 0.46). Their later study [122] further showed that functional connectivity with the hippocampus can forecast the severity of PTSD symptoms in adult subjects 6 months after injury (*n =* 98, *R =* 0.30). Their findings support the view that the hippocampus and amygdala are core regions in PTSD diagnosis. Despite the usefulness of hypothesis-driven methods for screening core regions for diagnostic classification, this method has not yet been applied to feature selection in OCD.

A potential difficulty in identifying core regions in OCD is the contradictory findings among case-control studies in which imaging techniques are commonly used to examine the neural mechanisms underlying this disorder. These inconsistent findings may result from small sample sizes, medication, and duration of illness. These studies provide valuable but noisy information that is difficult to integrate and translate into clinical advances. One way to systematically combine results from several studies and obtain a conclusion with greater statistical power to draw out core regions in OCD is meta-analysis [123]. Moreover, meta-analysis has been successfully implemented as a method of feature selection. Specifically, Dukart *et al.* [124] combined datasets from structural MRI and [F18] fluorodeoxyglucose PET to automatically detect AD based on the results of meta-analyses [125]. Features extracted from meta-analyses resulted in an accuracy of 88.0% in one dataset (AD = 28, HC = 28), and 91.0% in an independent cohort (AD = 21, HC = 13). In another study, Sundermann *et al.* [126] used meta-analysis to screen resting state studies and extract key features of MDD. In subsequent research, the authors used all pairwise functional connectivity among 38 meta-analytically defined brain regions as features to classify 180 patients with MDD from 180 HCs [127]. However, the SVM models achieved diagnostic accuracies around the chance level in the training dataset. As meta-analysis methods pool information from numerous studies, this approach could increase the statistical power and therefore reduce type II errors. However, the bias in small datasets might undermine the advantages of the meta-analysis for feature reduction. Future studies in a large cohort are encouraged to validate the power of meta-analysis in feature selection.

OCD is a highly heterogeneous disease with high levels of comorbidity [10]. The high heterogeneity in patients with OCD adds noise to the neuroimaging features, making it difficult to extract the key features. To solve this problem, the recruitment of “pure patients” with OCD who do not meet the criteria for other psychiatric disorders has been proposed. However, stricter criteria make recruitment more difficult. Animal models of OCD (especially the nonhuman primate model) provide an alternative way to harmonize individual differences. A common and clear etiology could suppress noise from individual differences and improve efficiency to identify core regions for diagnostic classification. To achieve this, Zhan *et al.* [31] adopted a cross-species feature selection framework for screening key regions related to stereotypic behaviors. This was done using transgenic monkeys with methyl-CpG binding protein 2 overexpressed in the brain, which results in autism-like behaviors [128]. First, the group least absolute shrinkage and selection operator (LASSO) algorithm was applied in the transgenic monkey dataset to extract vital brain regions for distinguishing transgenic monkeys from typically developing monkeys. Specifically, nine core regions were extracted: the left central temporal cortex, right superior temporal cortex, right dorsolateral PFC, right primary somatosensory cortex, right primary motor cortex, left ACC, right centrolateral PFC, left superior parietal cortex, and right ventrolateral PFC. Next, the identified brain regions were considered as seed regions to construct classifiers for classification in human datasets. The monkey-derived classifier successfully distinguished the ASD cohort from HC with high accuracy, outperforming the human-derived classifiers (ABIDE-I cohort, 82.1% *versus* 61.3%; ABIDE-II cohort, 75.2% *versus* 60.4%). This pioneering study demonstrated the advantages of animal models for reducing clinical heterogeneity and providing valuable key features for human datasets.

### Validation of Potential Core Regions in OCD

In animal models, a variety of techniques can be applied to up-regulate or down-regulate the activity in specific regions or circuits, such as optogenetics, chemogenetics, and deep brain stimulation [129, 130]. Animal models, therefore, provide a valuable platform in which to validate potential core regions in OCD. Hyperactivity in the OFC and striatum is tightly implicated in OCD, and successful treatments may normalize this hyperconnectivity [6]. Ahmari *et al.* [131] reported that repeated optogenetic excitation of the axon terminals of the OFC in the ventral striatum generated persistent grooming behavior, a well-known compulsive-like behavior in mice. Moreover, the elevated grooming evoked by chronic optogenetic modulation was reversed by fluoxetine. This study demonstrated the causal relationship between the OFC-striatal circuit and the pathophysiology of OCD. In mice, deletion of the *Sapap3* gene induces excessive grooming [132]. Using optogenetic techniques, Burguière *et al.* [133] found that stimulation of the lateral OFC and its terminals in the striatum alleviated the elevated grooming response during a conditioning task. In a recent study, Ramírez-Armenta *et al.* [134] applied optogenetics to inhibit the activity of the dorsomedial striatum in *Sapap3*-knockout mice and found that specific suppression of the striatal indirect pathway neurons rescued the excessive grooming. These findings set good examples to validate the causal role of brain regions in the pathophysiology of OCD. In the future, validation of other proposed core regions will advance our understanding of the circuits of OCD and the clinical translation of neuroimaging-based diagnosis.

## Perspectives

### Transdiagnostic Perspective on OCD

Although obsession and compulsion are core OCD symptoms, there is substantial OCD comorbidity with anxiety, depression, and other symptoms [10]. Moreover, obsession and compulsion are broadly observed in other psychiatric disorders, including addiction, autism spectrum disorder, generalized anxiety disorder, and eating disorder. To overcome this problem, a Research Domain Criteria strategy has been proposed to shift the focus away from classical diagnostic categories and symptoms to dissecting the neural circuits underlying the maladaptive behavior [4, 135]. This strategy requires a transdiagnostic view of psychiatric disorders to map brain-behavior relationships. Several studies have suggested that compulsion is the main candidate for OCD and related disorders [136], and research has tried to dissect the neural mechanisms underlying compulsive behaviors across different diagnostic disorders. For instance, Montigny *et al.* [137] applied a higher-order two-factor model to extract the compulsivity construct in a multisite adolescent dataset (*n* = 1938), and brain gray matter volume in the bilateral OFC, right ventral striatum, and right dorsolateral PFC was significantly linked to the compulsivity. In a large multi-site dataset acquired from adolescent subjects (*n* = 11876, 9–10 years old, subclinical-clinical population), Pagliaccio *et al.* [138] examined the associations between obsessive-compulsive symptoms (OCS) and brain structural and functional data. They reported a negative correlation between OCS scores and fractional anisotropy of the superior corticostriatal tract, and higher OCS scores were associated with lower connectivity within the dorsal attention network and lower anticorrelation between the dorsal attention network and the default mode network. In a small cohort of children and adolescents (ASD = 24, OCD = 25), Akkermans *et al.* [139] reported that higher functional connectivity between the left NAc and right premotor/middle frontal gyrus is associated with more compulsivity measured by repetitive behaviors. These studies have implied the existence of a continuum of compulsivity. Further studies are encouraged to apply machine learning algorithms to identify key features related to compulsivity. Other key dimensions of OCD, like anxiety and obsession, are also important directions to dissect the heterogeneity in patients with OCD and related disorders.

### From Classification to Prognostic Prediction

The search for neuroimaging biomarkers in the treatment of OCD is rapidly underway. Although several studies have tried to construct the relationship between baseline imaging data and treatment outcomes [140], machine learning algorithms have not been widely adopted [141–143]. In one of these studies, Reggente and colleagues [142] applied the LASSO regression algorithm to predict the treatment response to cognitive behavioral therapy based on baseline network connectivity patterns. They reported that the severity of OCD after treatment could be predicted by functional connectivity patterns within the default mode network and visual network, explaining up to 67% of the variance. Pagliaccio *et al.* [141] applied task-based fMRI data to predict the response to exposure therapy. The remission could be forecasted by brain activation within the cingulo-opercular and default mode network in the Simon spatial incompatibility task, specifically the anterior insula and anterior/posterior cingulate cortices. In future studies, it is promising to apply the features extracted from core regions in the diagnostic classification of OCD to the prediction of treatment outcomes.

## Conclusions

Cross-sectional studies in OCD have advanced our understanding of its pathophysiology. However, inefficient diagnosis and poor treatment remain important issues in the field. With the rise of machine learning and neuroimaging, diagnostic classification using imaging biomarkers is rapidly developing. Although the sensitivity and specificity of classification are not optimized, this direction might shed light on the data-driven individualized diagnosis. The main problem in diagnostic classification is how to extract effective features to boost the performance of the algorithms. Taking together neuroimaging findings on the neural correlates of OCD, neuromodulation treatments, and practices in diagnostic classification, we propose a classification framework based on “core regions” of the brain, which are indispensable areas among the mass features for classification algorithms. We further introduce hypothesis-driven or data-driven methods to screen these core regions from the whole brain. Advanced circuit modulation techniques offer opportunities to interrogate the potential core regions proposed in patients. Meanwhile, data from other levels also provide different angles to understand the role of core regions in the context of the pathophysiology of OCD, such as the spatial transcriptome [144] and metabolome [145] in the brain. Nevertheless, heterogeneity and comorbidity in patients diagnosed based on symptoms still hinder precise diagnosis and treatment. A transition from the diagnostic category to a transdiagnostic view is urgently needed to push the improvement of the whole field. Besides, applying machine learning to prognosis is an important direction to achieve the goal of precise medicine [146].
